# Draft Genome Sequence of *Staphylococcus chromogenes* Strain MU 970, Isolated from a Case of Chronic Bovine Mastitis

**DOI:** 10.1128/genomeA.00835-14

**Published:** 2014-08-14

**Authors:** Pamela R. Fry, Michael J. Calcutt, Mark F. Foecking, Hsin-Yeh Hsieh, Douglas G. Suntrup, Jeanette Perry, George C. Stewart, John R. Middleton

**Affiliations:** a Department of Veterinary Medicine and Surgery, University of Missouri, Columbia, Missouri, USA; b Department of Veterinary Pathobiology, University of Missouri, Columbia, Missouri, USA; c Bond Life Sciences Center, University of Missouri, Columbia, Missouri, USA

## Abstract

Coagulase-negative staphylococcal species are a common cause of subclinical bovine mastitis, with *Staphylococcus chromogenes* being one of the most frequently identified species in these cases. The draft genome sequence of an *S. chromogenes* isolate (MU 970) recovered from the milk of a cow with a chronic intramammary infection is reported here.

## GENOME ANNOUNCEMENT

*Staphylococcus chromogenes* is a Gram-positive, nonhemolytic, yellow-orange coccus. This organism was originally considered a subspecies of *Staphylococcus hyicus*; however, it is now classified as a separate species (1). *S. chromogenes* has been found to be the most prevalent coagulase-negative staphylococcal (CNS) species associated with subclinical intramammary infection (IMI) in dairy cattle in several studies (2–4). *S. chromogenes* has also been associated with persistent infections and elevated milk somatic cell counts (5). However, little is known about the genetic basis for pathogenesis of this organism. Hence, a draft genome sequence of *S. chromogenes* MU 970 was determined. This strain was isolated from the right rear mammary quarter of a Holstein cow at the University of Missouri Dairy (with Animal Care and Use Committee approval) for 16 consecutive months, between July 2005 and October 2006. The first and last isolate recovered from this IMI had identical pulsed-field gel electrophoresis banding patterns. The geometric mean somatic cell count associated with this IMI was 365,000 cells/ml.

Next-generation sequencing (454 Titanium) was performed at the Genome Institute, Washington University, St. Louis, Missouri, USA. Newbler assembly (Roche) of 178,110 reads resulted in 38 contigs (largest 558,330 bp; *N*_50_ 258 kb). The genome of 2,344,537 bp was covered at a sequencing depth of 24.7-fold and comprised 36.54% G+C. Auto-annotation of the contigs was performed at NCBI using the Prokaryotic Genome Annotation Pipeline (PGAP). A total of 2,311 open-reading frames (ORFs) and 60 tRNAs were annotated using GeneMarkS+. Five pseudogenes were verified through Sanger sequencing. Analysis of contig ends predicted that 6 RNA operons are present.

One plasmid sequence (19.3 kb) was identified in the genome of isolate MU 970. This plasmid contains an apparent transposable element with >99% identity to *Staphylococcus aureus* Tn*552*, a well-characterized 6.5-kb β-lactamase-encoding transposon (6), as well as a novel glycosyltransferase gene.

A gene predicted to encode a multidrug resistance efflux pump was identified. A phytoene dehydrogenase-encoding gene, with 67% identity to the *S. aureus* dehydrosqualene desaturase gene, was also found and is expected to contribute to the observed pigmentation.

Putative virulence factor genes recognized included *sasH*, which has previously been identified in *S. aureus* and determined to code for a cell wall–associated adenosine synthase that converts adenosine nucleotides into adenosine. Adenosine is a strong immunomodulator that helps staphylococci escape phagocyte-induced killing (7). Also, two biofilm-associated protein genes and two ORFs with 34% and 37% identity to the *S. aureus* fibronectin binding protein A were identified. This genome contains a gene encoding a putative superantigen-like protein, one gene encoding an IgG binding protein, and an ORF with 41% identity to a predicted coagulase of *Staphylococcus pseudintermedius*.

The data reported here represent the first *S. chromogenes* genome sequence available. These data should provide useful information for future studies on the pathogenesis of *S. chromogenes* IMI in the bovine, as well as studies to identify possible targets for therapeutic or vaccine interventions.

### Nucleotide sequence accession number.

This draft genome has been deposited at DDBJ/EMBL/GenBank under the accession number JMJF01000001.
